# A Case of Haemorrhagic-Onset Glioblastoma With Delayed Diagnosis

**DOI:** 10.7759/cureus.34672

**Published:** 2023-02-06

**Authors:** Mayuko Otomo, Masayuki Kanamori, Shiho Sato, Yoshiteru Shimoda, Mika Watanabe, Tomohiro Kawaguchi, Ryuta Saito, Teiji Tominaga

**Affiliations:** 1 Department of Neurosurgery, Tohoku University Graduate School of Medicine, Sendai, JPN; 2 Department of Diagnostic Radiology, Tohoku University Graduate School of Medicine, Sendai, JPN; 3 Department of Pathology, Tohoku University Hospital, Sendai, JPN; 4 Department of Neurosurgery, Kohnan Hospital, Sendai, JPN; 5 Department of Neurosurgery, Nagoya University Graduate School of Medicine, Nagoya, JPN

**Keywords:** idh-wildtype, brain mr images, delayed diagnosis, intracerebral haemorrhage, glioblastoma

## Abstract

Glioblastoma sometimes develops with acute onset due to intracerebral hemorrhage. Although it is sometimes difficult to diagnose patients with hemorrhagic-onset glioblastoma at the acute phase of intracerebral hemorrhage (ICH), the progressive enlargement of perifocal edema or the development of contrast-enhanced lesion triggers the diagnosis of glioblastoma within six months. Herein, we present a rare case of glioblastoma in which the diagnosis was delayed as long as 17 months after ICH. A 62-year-old man presented with a headache and aphasia. Computed tomography revealed ICH in the left temporal lobe. Magnetic resonance (MR) images revealed that the hematoma had a mix of isointense and surrounding hypointense lesions on T1-weighted MR images and gadolinium-enhanced lesions at the wall and the septum of the hematoma. An endoscopic evacuation of the hematoma was performed. No causative lesions were found during intraoperative and histological examinations. After seven months, abnormal signals were completely resolved on MR images, except for the small and stable enhanced lesion on three-dimensional gadolinium-enhanced T1-weighted MR imaging (3D Gd-T1WI) at the base of the hematoma, which did not change in size for seven months. However, a large gadolinium-enhanced lesion at the left temporal lobe developed 17 months after ICH. He underwent total resection of the lesion and was diagnosed with glioblastoma. He received radiation therapy and temozolomide but died of disseminated recurrence 31 months after ICH. In conclusion, this report presents a didactic case of glioblastoma in which the diagnosis of glioblastoma was delayed 17 months after ICH whereas hemorrhagic-onset glioblastoma was previously considered ruled out in cases in which six months or more have passed after ICH. In order not to overlook these cases, follow-up with 3D Gd-T1WI is essential in the case of suspected tumor-related ICH and close follow-up is recommended when the enhanced lesion does not resolve after a long period even if it does not grow.

## Introduction

Spontaneous intracerebral hemorrhage (ICH) is frequently associated with hypertension, excessive alcohol use, and cerebral amyloid angiopathy [1]. Because hemorrhage can also originate from an intracerebral tumor in 4.4%-7.2% of cases [2-4], the possibility of a hemorrhagic-onset intracerebral tumor should be ruled out at diagnosis. In clinical practice, radiological examination at ICH onset, macroscopic exploration, and histological examination at ICH evacuation can reveal the causative tumourous lesion for ICH [5,6]. However, it is sometimes difficult to diagnose patients with hemorrhagic-onset intracerebral tumors based on emergent computed tomography (CT) and magnetic resonance (MR) images or intraoperative endoscopic or microscopic sampling during the evacuation of the hematoma [7,8]. Therefore, patients with atypical ICH are evaluated over time through repeat CT or MR imaging even if no evidence of brain tumor on histological and radiological findings exists.

Glioblastoma is one of the pathologies that frequently can cause spontaneous ICH [3]. In patients with glioblastoma, diagnosis is sometimes delayed, similar to other intracerebral tumors. Even if the definitive diagnosis is not obtained at onset, hemorrhagic-onset glioblastoma is diagnosed based on the symptoms because the disease is progressive mostly within six months [7-12], reflecting rapid growth. Based on these reports, hemorrhagic-onset glioblastoma is considered ruled out in cases in which six months or more have passed since ICH.

This report presents a didactic case of glioblastoma in which the diagnosis of glioblastoma was delayed 17 months after ICH. Consent for the publication of this case report was obtained from his family, and this study was approved by the ethical board of Tohoku University Hospital (No. 23888).

## Case presentation

Clinical course

**Figure 1:**
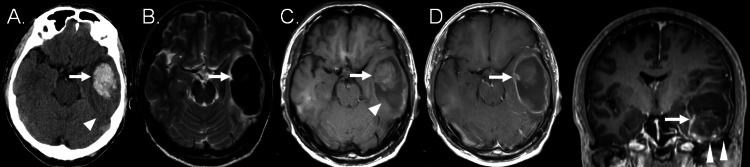
Computed tomography (CT) obtained at the onset and magnetic resonance (MR) images obtained on day five after intracerebral hemorrhage at the left temporal lobe. A: CT, showing mixed hyperdense (arrow) and surrounding isodense lesion (arrowhead) at the left temporal lobe. B–D: MR images, showing a homogenously hypointense lesion on T2-weighted MR image (arrow), (B) mixed isointense (arrowhead), and surrounding hypointense lesion (arrow) on T1-weighted MR image. (C) Peripheral linear and nodular (arrowheads) enhancement around the hematoma and septum (arrows) of the hematoma cavity enhanced on gadolinium-enhanced axial (left panel) and coronal (right panel) T1-weighted MR images. (D).

A 62-year-old man, previously diagnosed with hypertension, first complained of headache and aphasia. He was subsequently admitted to another hospital on day three. CT obtained at onset revealed mixed hyperdense and surrounding isodense lesions in the left temporal lobe (Figure 1A). He was diagnosed with left temporal metachronous ICH and received conservative treatment. However, his symptoms worsened gradually. Therefore, he was transferred to our hospital on day five. MR images obtained on day five revealed that this lesion was homogenously hypointense on T2-weighted MR images (T2WI) (Figure 1B) and mixed isointense and surrounding hypointense on T1-weighted MR images (T1WI) (Figure 1C). On 6-mm thick, peripheral linear and nodular enhancement around the haematoma and enhanced septum in the haematoma were noted (Figure 1D).

**Figure 2:**
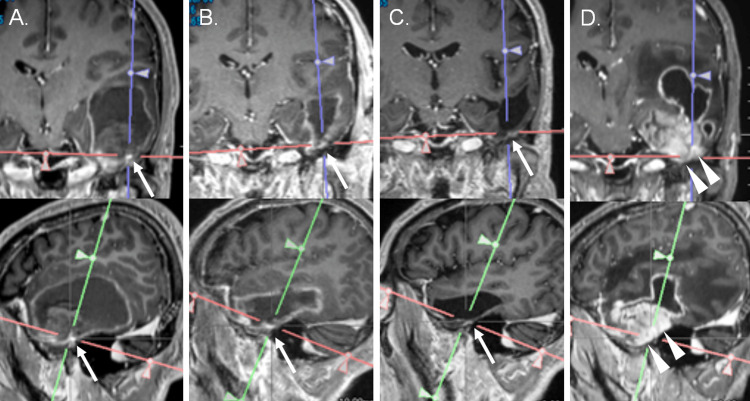
Magnetic resonance (MR) images demonstrating the enhancement around the hematoma and following glioblastoma, reconstructed from three-dimensional gadolinium-enhanced T1-weighted MR images on day five after intracerebral hemorrhage (ICH) (A), immediately after ICH evacuation (B), seven months after ICH (C) and 17 months after ICH (D). There were peripheral linear and nodular (center of the crossbar, arrows in A-C) enhancements on preoperative and postoperative MR images (A and B). The nodular lesion did not change in size and was still enhanced seven months after ICH (C), whereas peripheral linear enhancement disappeared. Irregular mass lesion (arrowheads in D) developed at the corresponding site of the nodular lesion at A–C.

The nodular lesions were located anteriorly and caudally of the hematoma on three-dimensional gadolinium contrast-enhanced T1WI (3D Gd-T1WI) (Figure 2A). From these findings, he was diagnosed with metachronous hematoma at the left temporal lobe due to an intracerebral tumor. To relieve his symptoms and explore the cause of the hemorrhage, endoscopic evacuation of the left temporal hematoma was conducted on day six (Figure 2B). The hematoma consisted of fluid hematoma and coagulant hematoma at the tip of the temporal lobe. However, no abnormal tissues were found in the hematoma cavity. For further analysis, tissue for histological examination was obtained from the lateral and caudal walls around the temporal base, where nodular enhanced areas were located, respectively, in the hematoma’s cavity and from the coagulant hematoma. However, no neoplastic lesions were observed in the histological material. Since we could not rule out the occult hemorrhagic lesion from the finding of the mixed intense hematoma and residual peripheral linear and nodular enhancement, he was followed up using serial MR images. However, nodular lesions, which were strongly enhanced at onset and immediately after evacuation of hematoma, were still enhanced without enlargement for seven months (Figure 2C). Although contrast-enhanced findings on CT have been reported to persist for more than three months [13], there is no report on the duration of gadolinium enhancement on MR imaging around the hematoma. Therefore, we could not rule out the presence of a tumour or small arteriovenous malformation at the residual enhanced lesion, so we scheduled follow-up 12 months thereafter. Before the scheduled appointment, he developed drowsiness and deterioration of aphasia 17 months after the onset of temporal ICH. 3D Gd-T1WI also revealed an irregularly enhanced lesion at the left temporal lobe (Figure 2D). The spatial relationship between the small nodular lesion and the irregular large mass suggested that glioblastoma could develop from the residual nodular lesion (Figures 2C, 2D).

**Figure 3:**
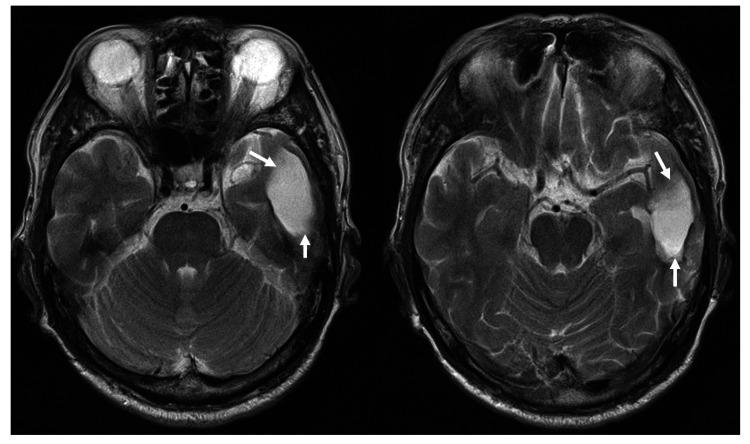
Magnetic resonance (MR) images seven months after the onset of intracerebral hemorrhage. No abnormal lesion signal around the hematoma’s cavity (arrows) were found on T2-weighted MR images.

Seven months after the surgery, the hyperintense area in the cerebral parenchyma on T2WI was completely resolved (Figure 3).

**Figure 4:**
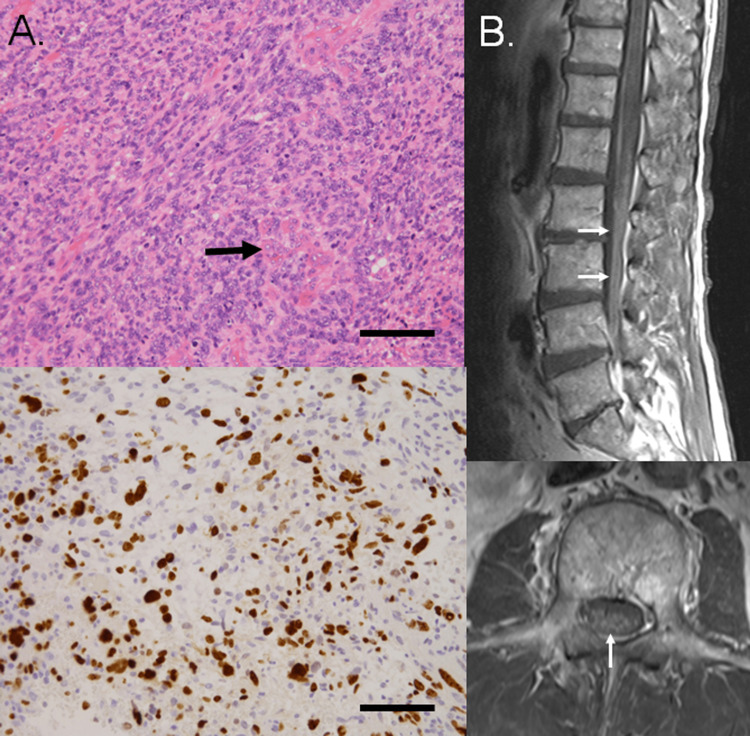
Histological findings of glioblastoma and spinal magnetic resonance (MR) images at recurrence. A. Haematoxylin–eosin staining (upper panel) showing atypical glial cells with marked nuclear atypia pleomorphism, proliferating at high cellularity. Microvascular proliferation (arrow) was observed. On immunohistochemical examinations (lower panel) for Ki67, the Ki67 labeling index was 30.0%. Bar = 0.1 mm. B. Sagittal spinal and axial MR images obtained 28 months after intracerebral hemorrhage, showing dissemination around the cauda equina (arrows).

Consequently, he underwent left frontotemporal craniotomy and tumor removal. On histological examination, relatively small atypical glial cells, showing marked nuclear atypia and pleomorphic features, proliferated with high cellularity. Microvascular proliferation (Figure 4A) and pseudo-palisading necroses were also found. On immunohistochemical examination, tumor cells were negative for IDH R132H, and the Ki67 labeling index was 30.0% (Figure 4A). From these findings, the histological diagnosis was IDH wild-type glioblastoma [14]. Therefore, the patient received a concomitant 60 Gy radiation therapy at the local site and temozolomide, followed by adjuvant temozolomide. However, he presented dysuria and dysesthesia of his left leg due to spinal dissemination 28 months after ICH (Figure 4B). Unfortunately, he died of a progression of the disease 31 months after ICH.

## Discussion

To the best of our knowledge, this case of hemorrhagic-onset left temporal glioblastoma, IDH wild-type, had the longest latency from hemorrhage to the diagnosis of glioblastoma. He was initially diagnosed with atypical ICH at the left temporal lobe and we presumed that he had causative pathology including malignant glioma, metastatic tumor, or cavernous malformation. Unfortunately, preoperative imaging, intraoperative finding, histological examination, or serial MR imaging by seven months could not detect the underlying glioblastoma in this case.

In a report from a single institute and literature review of hemorrhagic-onset glioblastoma [7,8], five (28%) of the 18 adult patients were diagnosed on the same day with ICH onset and three (17%) were diagnosed within 14 days. They were diagnosed through radiographic examinations that were obtained at the acute phase of ICH or during histological examinations during ICH evacuation. Eight (44%) cases were then diagnosed 1-6 months from the onset of ICH. Most of them had progressive symptoms and radiographic findings, which were finally diagnosed as glioblastoma. Compared with those previous reports, our case had an extraordinarily long latent period from ICH onset to glioblastoma development. The true mechanisms for the extremely late development of glioblastoma after ICH onset remain unclear. A possible mechanism is a contrast-enhanced lesion that was not growing for seven months after ICH. Soto et al. reported a similar case, in which a small nodular enhanced lesion, which was stable for two months after temporal ICH, progressed rapidly after 4 months [7]. In our and their cases, the growth of glioblastoma might be suppressed transiently after the hemorrhagic event [12]. Alternatively, it cannot be ruled out that hematoma may have triggered gliomagenesis in the same way that traumatic brain contusion can cause glioblastoma [15,16]. However, the latter mechanism appeared unlikely due to the atypical ICH at onset, the latent period of cerebral contusion-induced glioblastoma for most cases of five years or more, or no reports of basic research or clinical practice to date.

Findings of the mixed intensity appearance of hematoma on T1WI and T2WI, delayed evolution pattern of the hyperintense area on T1WI, presence of irregular and discontinuous hemosiderin rim, and peripheral linear and nodular enhancement on Gd-T1WI suggest the ICH from the tumor [17,18]. However, detecting the lesion is difficult, especially at the acute and sub-acute phases because of the enhancement of hematoma and surrounding gliosis. To overcome this problem, the usefulness of dual-energy CT and MR perfusion has been reported [19,20]. However, in the present case, the nodular lesion was too small to be detected on MR perfusion (data not shown) at ICH onset and only 3D Gd-T1WI detected the putative origin of glioblastoma. From the clinical course in our case, we recommend repeating MR imaging including 3D Gd-T1WI after the disappearance of the enhancement of the gliosis around the hematoma in cases with suspected tumor-related hemorrhage to detect the small lesions.

We did not consider the residual enhanced lesion at seven months after ICH as glioblastoma because the lesion did not grow for seven months at that time. However, judging from the spatial correlations between the small enhanced lesions and progressive disease, the small enhanced lesion in Figure 2C should have been diagnosed as small and static glioblastoma. To avoid the delayed diagnosis, we should have continued vigorous follow-up of the residual small enhanced lesion, or undergone other diagnostic tests including methionine positron emission tomography.

## Conclusions

In conclusion, this report presents a didactic case of glioblastoma in which the diagnosis of glioblastoma was delayed 17 months after ICH whereas hemorrhagic-onset glioblastoma was previously considered ruled out in cases in which six months or more have passed after ICH. In order not to overlook these cases, follow-up with 3D Gd-T1WI is essential in the case of suspected tumor-related ICH and close follow-up is recommended when the enhanced lesion does not resolve after a long period even if it does not grow.
